# The World Health Organization African regional training course for mid-level immunization managers: lessons and future directions

**DOI:** 10.11604/pamj.2020.37.194.26295

**Published:** 2020-10-29

**Authors:** Balcha Masresha, Carine Dochez, Alice Bumgarner, Nathan Pienkowski, Richard Mihigo

**Affiliations:** 1 World Health Organization, Regional Office for Africa, Brazzaville, Congo,; 2 Network for Education and Support in Immunization, University of Antwerp, Antwerp, Belgium,; 3 Bull City Learning™, Durham, North Carolina, United States of America

**Keywords:** Immunization, vaccine preventable diseases, training, capacity building, mid-level managers, MLM, Africa

## Abstract

The World Health Organization (WHO) African regional training course for mid-level managers (MLM) of immunization programs launched in 2004, has undergone revisions across the years, to accommodate new developments in the field. In 2016, the WHO African regional office conducted a thorough review of the course materials and delivery methods to document lessons to help improve the course. Some of the gaps included inappropriate selection of trainees, inadequate focus on skills development, heavy reliance on text and presentations, as well as resource limitations to reach a critical mass of learners. The regional office worked with Bull City Learning to redesign the course materials along carefully crafted course objectives and curricula, and to assist facilitators to better deliver the course. In addition, the materials were converted into online learning tools. Within 10 months, a total of 3011 learners were enrolled in the online MLM training platform and earned a total of 9209 certificates. The MLM course will continue to be highly relevant as the immunization area of work expands significantly, with the addition of new vaccines, introduction of new technologies, and expanding opportunities for online learning.

## Introduction

Health worker training is an essential component of capacity building and strengthening health systems. The World Health Organization (WHO) plays an important role in terms of setting the norms and standards for public health measures, in terms of providing technical and operational guidelines as well as training for public health professionals and frontline health workers [1]. Within the immunization program, it has long been recognized that high staff turnover, and rapidly evolving programmatic contexts require frequent and regular training of service providers, as well as clinicians and immunization program staff. The regional strategic plan on immunization identifies the need for training as a means to build individual and institutional capacity to adequately plan, implement and monitor immunization programmes [2].

## Project evaluation

The WHO mid-level managers´ (MLM) immunization program training course was first developed in 2000, to complement the “Immunization in Practice” set of modules for immunization service providers. The African regional office of the WHO developed a regional MLM course in 2004, targeted towards immunization program managers at national and subnational levels. The course aimed to provide critical up-to-date technical information and outline the essential managerial principles to help program staff at different levels to manage the immunization program on a day-to-day basis. The course was designed to cover topics ranging from planning immunization programs to improving coverage, monitoring and evaluation [3, 4].

In the years since the launch of the regional MLM course, the global policy guidance on immunization has evolved, and new antigens are being introduced into national immunization schedules. In 2004, thirty of the 46 countries in the African region reported administrative coverage on 6-8 types of antigens in their immunization programs. However, by the end of 2019, 32 countries have at least 13 antigens in their immunization programs, and routine vaccination service is now provided as a scheduled service beyond the infant age group, to toddlers, older children and adolescents [5]. Countries have also been introduced to updated technological tools to support cold chain management and monitoring [6]. In the last two decades, the intensive support to routine immunization, the introduction of new vaccines, as well as the efforts towards the eradication of polio, the elimination of yellow fever and meningitis A epidemics, as well as the elimination of measles and neonatal tetanus have provided relevant lessons on the management and implementation of immunization within the African context [2, 7, 8].

The management of immunization programs and the provision of good quality services requires adequate trained workforce, with access to appropriate guidance and tools, as well as the necessary supervisory support to contribute towards the program goals. Various studies and national program reviews have indicated programmatic gaps that require strengthened managerial skills at national and subnational levels [9-13]. In addition, multi-country evaluations have demonstrated inadequate quality of training and supervision of managers as one of the root causes of suboptimal immunization program performance, indicating the need for more effective and targeted health worker training [14]. The role of health worker training in improving program performance and motivation is well documented [3, 15-19]. Since the introduction of the MLM course in the Region, numerous training workshops have been held at regional and sub-regional levels to develop a pool of trainers. In addition, nearly all countries in the region have conducted national and sub-national training sessions at different times in order to build the capacity of immunization program staff at different levels.

**Key lessons learnt:** the African regional MLM course materials have been modified a few times since 2004 in order to update the modules with new information reflecting the developments and operational experiences in the field. In 2016, the African regional office of the WHO critically examined the contents and the delivery of the course, with a view to document the lessons learnt and identify ways to improve its usefulness. Some of the major observations and lessons that eventually led to the complete revision of the MLM course included the following:

**Selection of trainees *:*** the MLM course is designed for trainees that already have some technical knowledge and operational/managerial experience. However, the selection of trainees was not always done targeting mid-level managers from the national and subnational levels. The inclusion into the course of academicians and personnel with no program management experience reduced the impact of the course.

**Course content *:*** the principal objective of the course needs to be to build problem-solving skills, as well as skills for planning, using data for decision making and day-to-day management of the program. The course materials and modes of delivery relied heavily on text and power-point presentations, which emphasize imparting knowledge over building the skills of the participants. For adult learners, the training was not adequately engaging.

**Pedagogical methods *:*** when the MLM course was cascaded to the subnational levels, there was inadequate guidance to trainers on the pedagogical methods to apply and on the standards for MLM training, often leading to inconsistent approaches and outcomes.

**Delivery method *:*** the training was designed to be delivered in a classroom approach, was resource intensive and created a limit to the number of people who could be trained at any one time. Hence it was not possible to do the training frequently enough to reach a critical mass of mid-level managers. The course was packaged as a 10 days´ complete course consisting of 16 modules, and so was time consuming. It was often not possible to tailor the training to individual country needs. In addition, the course was not making use of available technology and online platforms for training, including adopting self-paced online training as an option to make the materials even widely available.

**Revision of the MLM course materials:** based on these lessons, in 2017-2018, the WHO regional office for Africa worked with instructional design experts from Bull City Learning (N Carolina, USA) in order to redesign the MLM course modules with a view to increase the impact of the course. The revision involved critically looking at the course objectives and re-designing the curriculum map alongside the reformulated objectives, revisiting the module content and rewriting parts of the module to fit the objectives. Additional interactive exercises were developed as part of the focus on the skills development aspect of the training, the format of the modules was reorganized, and highly engaging visuals were included in the training materials. Fourteen modules were developed as part of the revision, each of which consisted of the facilitator guide, the participant resource book, the participant workbook, the facilitator answer book, the power point training deck, as well as additional materials for facilitation. The modules were organized in such a way that they could be used as learning tools independently, rather than as a package of multiple modules.

The MLM course is designed for health workers involved in the management of immunization programs and is complementary to the “Immunization in Practice” course aimed at the service delivery level and the “Vaccinology” courses aimed at higher level professionals and academicians. In order to get optimal results from these courses, it is imperative that the profile for the trainees of each of these courses is respected. Therefore, the revised MLM course puts emphasis on the careful selection of participants. The updated modules were piloted in a series of workshops in Sierra Leone and Ghana in 2018, and the necessary amendments were incorporated to improve the quality of the modules and to assist the facilitation process. In November 2019, a regional Training of Trainers (ToT) was conducted in Ethiopia using the new MLM course materials, involving 38 Anglophone participants. The 14 MLM modules were later translated into French and made available by March 2020. A regional francophone Training of Trainers was not possible due to the COVID pandemic. The Portuguese translation is expected to be available by the first quarter of 2021.

Alongside the translation of the course materials, the MLM training materials were modified for online self-paced training and released in November 2019, with the French versions following in July 2020. The development of the online modules made the training accessible freely and widely. Learners begin the course any time they choose, work on any modules they´re interested in, and complete them at their own pace. Learners are required to create an online account to take the MLM courses and must first pass an assessment in order to earn certification. As the lockdowns to limit COVID spread were applied in many countries starting in mid-March 2020, we noted a huge demand for the online courses, with a rapid expansion of the number of users in the subsequent months. From 1^st^ November 2019 to 31^st^ August 2020, a total of 3011 learners were enrolled in the online MLM training platform and earned a total of 9209 certificates. The majority of these learners (94%) came from 41 countries in the African region in addition to participants from 30 countries beyond the Region. Among these learners, 408 of them earned master certification after successfully completing all 14 modules [20].

**The way forward:** the MLM course continues to be highly relevant in the future for many reasons. The goals and targets adopted for the elimination of various vaccine-preventable diseases (VPDs) at global and national levels are ambitious and require sustained capacity building. The immunization area of work is getting more and more complex, with more underutilized and new antigens being made available, expanding vaccination schedules, increasing logistical challenges, as well as vast amount of resources and program costs being involved. At the same time, there is still a continued high turnover of immunization program staff at country level. Moreover, there is increasing focus on assuring vaccine safety, and a need to address issues related to the growing vaccine hesitancy in the region. The technologies for cold chain and vaccine management, as well as vaccine delivery are changing. With the expanding access to internet across Africa, there is a huge potential and a growing interest in online learning which creates a perfect opportunity to continue to offer highly relevant self-paced online courses. As more and more countries adopt the introduction of scheduled doses of antigens in the second year of life, in early childhood and during adolescence, a new module is currently being developed to address programmatic issues related to the expanding age target for vaccination.

The implementation of immunization mid-level managers´ trainings at national level will need to be guided by a regular, structured and standardized training needs assessment. National immunization programs will need to set the balance between workshop-style training facilitated by subject matter experts, and the self-paced online courses. Countries will have to carefully select participants for the immunization mid-level managers´ courses, while making sure that others are referred to the training courses more suited to their needs. In addition to the national immunization programs and the partner agencies, it will be important to develop a pool of trainers from academic institutions and centers of excellence so that there is a critical mass of trainers who can assist in the delivery of MLM and other in-service trainings. There is a need to integrate post-training follow-up and supportive supervision in order to ensure that the learnt skills are retained and appropriately utilized in the field setting. Such follow-up may also be conducted remotely through mobile networking applications, with the integration of microlearning to complement on-the-job and in-person encounters. And finally, countries should embed regular monitoring of health worker knowledge and practice as well as the measurement of training outcomes into their program monitoring in order to timely identify and address any knowledge and skill gaps.
